# The effects of exercise on COVID-19 therapeutics: A protocol for systematic review and meta-analysis

**DOI:** 10.1097/MD.0000000000022345

**Published:** 2020-09-18

**Authors:** Zhangmeng Xu, Yong Chen, Duoduo Yu, Dongdong Mao, Ting Wang, Donghong Feng, Tao Li, Shengsong Yan, Yaming Yu

**Affiliations:** a Chengdu Sport University; b Sichuan Province Orthopaedic Hospital; c Hospital of Chengdu University of Traditional Chinese Medicine; d Chengdu University of Traditional Chinese Medicine, Chengdu, Sichuan Province, China.

**Keywords:** COVID-19, exercise, systematic review

## Abstract

**Background:**

At the end of 2019, peoples normal lives were disrupted by a sudden plague (COVID-19), the huge impact of COVID-19 on society has never been appeared. How to effectively prevent and treat COVID-19 is a concern for all health care workers. Exercise as a green and cheap complementary therapy, which has been proven to improve the immune capacity of the body and prevent infection. The main purpose of this study is to provide a reliable methodological guidance and credible evidence for exercise on COVID-19 therapeutic.

**Methods:**

This protocol is guided by the Preferred Reporting Items for Systematic Reviews and Meta-Analyses Protocols. We will search the following database sources for the Randomized controlled trials: the Cochrane Library, PubMed, EMBASE, Web of Science, Chinese Biomedical Literature Database (CBM), Chinese National Knowledge Infrastructure Database (CNKI), Chinese Science and the Wanfang Database. All randomized controlled trials of exercise therapy for COVID-19 in the above database will be considered for inclusion, and high-quality articles will be screened for data extraction and analysis, to summarize the therapeutic effect of exercise on COVID-19 patients.

**Results:**

In this study, we hope to find strong evidence for the treatment of COVID-19 by exercise.

**Conclusion:**

The conclusion of our study will provide credible evidence to judge whether exercise is an effective intervention on the COVID-19 patients therapeutic, and guide future researches.

PROSPERO registration number: CRD42020200883.

## 1 Introduction

On December 31, 2019, Wuhan Municipal Health Committee reported 27 cases of unexplained pneumonia. After study by Chinese health experts, the pathogen of unexplained pneumonia was identified as a novel coronavirus on January 9, 2020, now known as severe acute respiratory syndrome coronavirus 2 (SARS-CoV-2). Subsequently, SARS-CoV-2 began an epidemic in China, and more than 80,000 Chinese residents were infected with SARS-CoV-2. On February 11, 2020, World Health Organization (WHO) officially named the pneumonia caused by SARS-CoV-2 as Corona Virus Disease 2019 (COVID-19). Subsequently, on March 11, WHO identified the COVID-19 outbreak characteristically as a pandemic. In the next 5 months, COVID-19 began to spread around the world, more than 20 million people infected with COVID-19 and more than 700,000 deaths worldwide by August 12, 2020, and over 210 countries and territories are involved.

SARS-CoV-2 is one of the coronaviruses. Coronavirus is a positive single-stranded RNA virus with a diameter of 80 to 120 nm, which can be divided into 4 genera, namely α, β, δ, and γ. The SARS-CoV-2 is β-coronavirus, and it is the seventh coronavirus have found that can infect human. Current studies have found that people susceptible to SARS-COV-2 include of all ages, and the main source of infection are confirmed COVID-19 patients, and those in the incubation period and asymptomatic infected persons.^[1]^ SARS-CoV-2 has 4 main ways of transmission.^[2,3]^ First is droplet transmission, general people caused by inhalation droplets that is emitted by infected person when are coughing or talking. Second is transmission by close contact, that is contact with the mucous membrane or damaged skin of the COVID-19 patient or the virus carrier person, or contact with the droplets of the infected person who left on the surface of the object; the third is aerosol transmission, that is inhaling aerosol formed of droplets which emitted by infected people; the fourth is other possible means of transmission, including fecal-oral transmission etc. The main initial symptoms of COVID-19 patients include fever, dry cough, fatigue, and few people accompanied with pharyngeal pain, muscle soreness, nasal congestion, and runny nose, etc. The early clinical symptoms of COVID-19 are similar to common influenza and without specificity. Most people with COVID-19 experience are mild to moderate illness, but around 15% progress to severe pneumonia, and about 5% of patients progress to acute respiratory distress syndrome.^[4]^ Studies have shown that among the many critical patients with COVID-19, the proportion of the elderly is higher, which is closely related to the relatively low immunity of the elderly.^[5,6]^

As a kind of green and cheap complementary therapy, exercise has a positive effect on many diseases. In 2007, American College of Sports Medicine and the American Medical Association officially proposed “Exercise is Medicine”. Studies have shown that exercise is closely related to the immune ability of the body.^[7]^ Regular exercise can significantly improve the immune ability of the body and reduce the incidence of infectious diseases,^[8]^ while long-term high-intensity exercise or lack of exercise is an important cause of immune decline.^[9,10]^ Bernardi and Simpson found that regular moderate intensity training can significantly improve the immune ability of the body and effectively reduce the incidence of respiratory diseases.^[11,12]^ Nieman and Grande also found that regular moderate-intensity physical activity can reduced incidence, duration and severity of upper respiratory tract (predominantly viral) infections.^[13,14]^ Shimojo et al study found that 1-hour swimming per day can attenuate serum levels of inflammatory cytokines and increased anti-inflammatory cytokines of mice with endotoxemia.^[15]^ Prospective studies have consistently shown that regular physical activity reduces the risk of infection^[16]^ and the burden of latent viral infections.^[17]^ In addition to improving immunity, regular exercise will also help to weight control, mood improvement, better sleep, and relieve anxiety,^[18]^ which are common concomitant symptoms in COVID-19 patients. Therefore, in this study, we aim to systematically review the positive role of exercise in promoting recovery of patients with COVID-19 and improving the quality of life of the COVID-19 cured population.

## 2 Method

### 2.1 The registration

In accordance with the guidelines, this systematic review protocol was registered with the International Prospective Register of Systematic Reviews (PROSPERO) on July 27, 2020 (registration number CRD42020200883). The consent of this protocol report is based on the Preferred Reporting Items for Systematic Review and Meta-Analysis Protocols (PRISMA-P) 2015^[19]^ statement guidelines.

### 2.2 Inclusion criteria for study selection

#### 2.2.1 Study designs

We will include researches related to exercise therapy of patients suffering from COVID-19. Due to language restrictions, we will search for articles in English and Chinese. In order to get a more objective and true evaluation, all articles must meet the following 4 conditions at the same time:

1. Published documents with complete documents data;
2. Participants were confirmed to have COVID-19;
3. The type of trial is randomized controlled trial (RCT);
4. The intervention group received exercise intervention for at least 2 months.

#### 2.2.2 Participants

We will include all patients who suffering from COVID-19 regardless of sex, age, racial group, education, and economic status. Pregnant women, postoperative infections, psychopaths, patients with severe pneumonia or other reasons who cannot exercise, patients with severe cardiovascular and/or liver and/or kidney diseases will not be included.

#### 2.2.3 Interventions

The studies at least one of the groups received exercise intervention will be included. Exercise intervention include aerobic exercise, daily exercise, endurance training, high-intensity interval training, resistance, exercise. Other stimulation methods such as postoperative rehabilitation training, sports rehabilitation after fracture and athlete training will be excluded.

#### 2.2.4 Comparators

This systematic review will include studies comparing exercise to non-exercise intervention controls (e.g., usual or standard care, relaxation), studies comparing different exercise or physical activity protocols (e.g., aerobic exercise versus resistance exercise), and studies comparing an exercise-based intervention to another treatment approach (e.g., pharmaceuticals, acupuncture).

#### 2.2.5 Outcomes

Primary outcomes: Time of disappearance of main symptoms (including fever, fatigue, cough, and temperature) recovery time, and serum cytokine levels. Secondary outcomes: Accompanying symptoms (such as myalgia, expectoration, stuffiness, runny nose, pharyngalgia, inhalation, chest distress, crackles, headache, nausea, vomiting, anorexia, diarrhea) disappear rate, CT image improvement, the results of COVID-19 nucleic acid test are negative on 2 consecutive occasions (not on the same day), average hospitalization time, occurrence rate of common type to severe form, clinical cure rate, and mortality.

### 2.3 Data sources

The following electronic databases will be searched from inception to July 2020: the Cochrane Library, PubMed, EMBASE, Web of Science, Chinese Biomedical Literature Database (CBM), Chinese National Knowledge Infrastructure Database (CNKI), Chinese Science and the Wanfang Database. To ensure literature saturation, we will scan the reference lists of included studies or relevant reviews identified through the search. We will also search the authors personal files to make sure that all relevant material has been captured.

### 2.4 Search strategy

The search terms on PubMed are as follows: exercise (e.g., “sport” or “activity” or “movement” or “train”); COVID-19 (e.g., “Corona Virus Disease 2019” or “Novel Corona Virus”); convalescence (e.g., “rehabilitation” or “recovery” or “decubation”); randomized controlled trial (e.g., “randomized” or “randomly” or “clinical trial”). Combinations of Medical Subject Headings (MeSH) and text words will be used. The same search term is used in other electronic databases. These search terms are shown in Table 1.

**Table 1:**
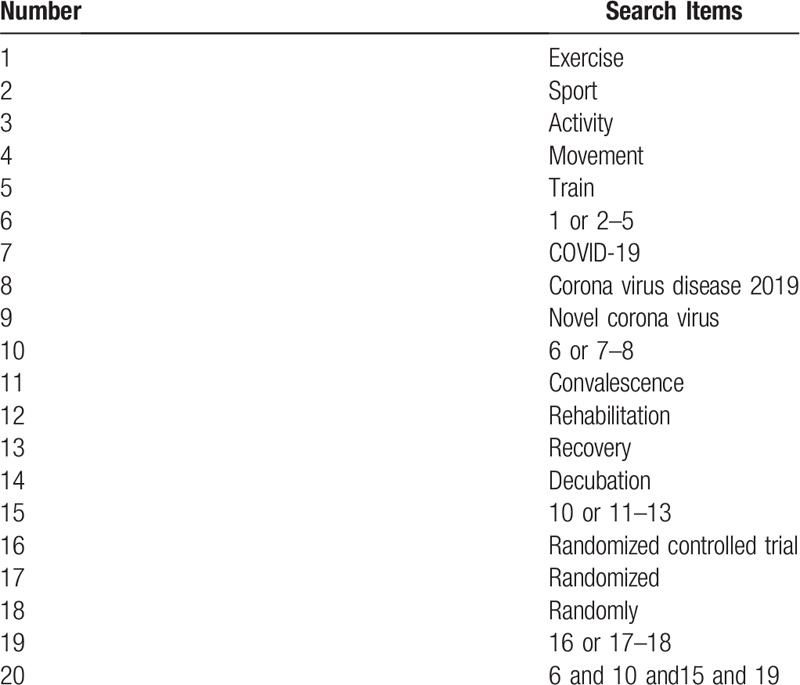
Search strategy for the PubMed database.

### 2.5 Data collection and analysis

#### 2.5.1 Selection of studies

**Figure 1:**
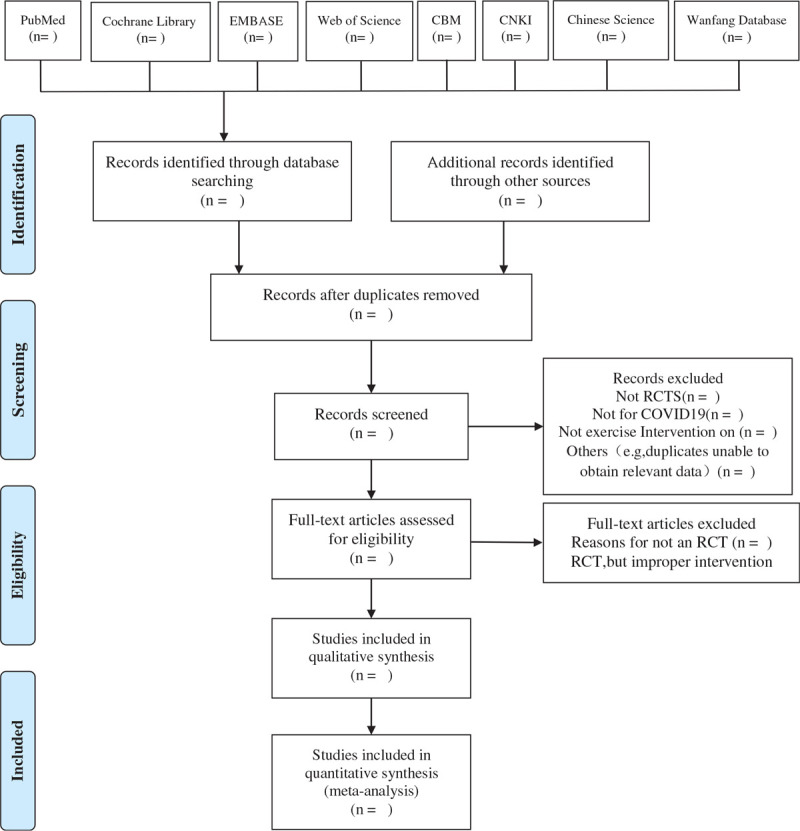
Flow chart of the study. Adapted from Preferred Reporting Items for Systematic Reviews and Meta-Analyses Protocols (PRISMA-P).

We chose the PRISMA flow chart to show the process of selecting literature for the entire study (Fig. 1). Before searching the literature, all reviewers will discuss and determine the screening criteria. After the screening requirements are clearly defined, the 2 reviewers (ZMX and YC) will independently review and screen the titles and abstracts yielded by the search against the inclusion criteria. In order to get qualified studies, we will then screen the full text reports and decide whether these meet the inclusion criteria and then excluded some duplicate studies or studies with incomplete information. The obtained literature will be managed by using EndNote software V.X8 (Thomson Corporation, United States). Any inconsistency is resolved by discussing with the third investigator.

#### 2.5.2 Data extraction and management

The reviewers will assess the eligibility of the studies independently using the inclusion and exclusion criteria. The following data then will be extracted from the studies selected for inclusion using a data collection form, and recorded onto an excel file: first author and year of publication, study design, participants (e.g., patients average age, gender, weigh, main symptoms and duration, underlying diseases), interventions (e.g., type and duration and frequency of exercise), outcomes (e.g., disappearance time of main symptoms, cure rate, incidence of severe pneumonia, mortality, average hospitalization time), adverse reactions, risk of bias assessment and funding sources. Three teams of reviewers will extract data independently and in duplicate from each eligible study, any disagreements will be resolved by discussion, with any ongoing differences in opinion being arbitrated by a third reviewer (YMY). We may also contact the authors of primary studies by telephone or email to ask for additional relevant information, if necessary. All data will be transferred into Review Manager Software (RevMan V.5.3) for analysis and synthesis.

#### 2.5.3 Assessment of risk of bias in included studies

Two authors (DDM, DDY) independently assessed the methodological quality of each trial according to the standards advised by the Cochrane Handbook For Systematic Reviews of Interventions. Any disagreements were resolved by discussion and reached consensus through a third reviewer (YMY). The risk of bias was evaluated for each study by assessing the randomization process, the treatment allocation concealment, blinding of participants and personnel, blinding of outcome assessment, the completeness of the data and selective outcome reporting or not. A judgment as to the possible risk of bias on each of the 6 domains will be made from the extracted information, the risk of bias is evaluated at 3 levels, namely, low risk, high risk, and ambiguity. If the information is vague, we will try to contact the corresponding author of the article.

#### 2.5.4 Measures of treatment effect

In this protocol, we will use 95% confidence interval (CI) risk ratio (RR) to rigorously analyze the dichotomous data. And for the continuous data, weight mean difference or standard mean difference is used to measure the efficacy of 95% CI. Skewed data and non-quantitative data will be presented descriptively.

#### 2.5.5 Unit of analysis issues

Preliminary analysis will be conducted by random grouping of individual participant. However, special problems often arise in studies that are not standard designed, such as cluster randomized trials or studies with multiple treatment groups. For clustered randomized trials, we will refine the results by extracting an interclass correlation coefficient based on the Cochrane Handbook For Systematic Reviews of Interventions. And for multiple treatment groups, we will present the additional treatment arms. Where the additional treatment arms are not relevant, they will not be taken into account.

#### 2.5.6 Management of missing data

We will try our best to ensure the integrity of the data. When there are missing data, we will try our best to contact the corresponding author of the article, including but not limited to sending emails or making a phone call. If the corresponding author cannot be contacted, we will use sensitivity analysis to assess the impact of the missing data on the final outcome, if the impact on the outcome is significant, we will remove the experiment with incomplete data. After data integrity is assured, intention analysis therapy and sensitivity analysis will be performed.

#### 2.5.7 Assessment of heterogeneity

For the detection of statistical heterogeneity, the Chi^2^ test (significance level: 0.1) and *I*^2^ statistic will be used to test the heterogeneity among trials. When the *I*^2^ value is <40% means might not be important; 30%< *I*^2^ < 60% means represent moderate heterogeneity; 50%< *I*^2^ < 90% means substantial heterogeneity; 75%< I^2^ < 100% means considerable heterogeneity. If high levels of heterogeneity among the trials exist (*I*^2^ ≥ 50% or *P* < .1), the study design and characteristics in the included studies will be analyzed and subgroup analysis or sensitivity analysis will be try to use to explain the heterogeneity.

#### 2.5.8 Data synthesis

Each outcome will be calculated and combined using the RevMan 5.3. Specific implementation was based on the current version of the Cochrane Handbook for Systematic Reviews of Interventions. If tests of heterogeneity are not significant, the Mantel-Haenszel method will be chosen for fixed effect model, and if statistical heterogeneity is observed (*I*^2^ ≥ 50% or *P* < .1), the random effects model will be used. If heterogeneity is substantial, we will perform a narrative, qualitative summary.

#### 2.5.9 Assessment of reporting biases

In this analysis, once >10 trials are included, funnel plots could be used to test for reporting bias.

#### 2.5.10 Subgroup analysis

It is possible that individual studies may consist of multiple treatment groups, subgroup analysis will be performed to explain heterogeneity if possible. Factors such as following will be considered:

1. Patients characteristics (age, sex, underlying diseases).
2. Types of interventions (aerobic exercise, daily exercise, endurance training, high-intensity interval training, resistance, exercise ect.).
3. Duration and frequency of exercise.

#### 2.5.11 Sensitivity analysis

Sensitivity analysis will be performed according to sample size, study design, heterogeneous quality, methodological quality and statistical model, the trials with quality defects will be excluded to ensure the stability of the analysis results.

#### 2.5.12 Grading the quality of evidence

This paper will use the evidence quality rating method to evaluate the results obtained from this analysis. GRADE will be assessed across the domains of risk of bias, consistency, directness, precision and publication bias. In the context of the system review, quality reflects our confidence in the effectiveness of assessment. It has 4 evaluation levels, namely, high (further research is very unlikely to change our confidence in the estimate of effect), moderate (further research is likely to have an important impact on our confidence in the estimate of effect and may change the estimate), low (further research is very likely to have an important impact on our confidence in the estimate of effect and is likely to change the estimate), or very low (very uncertain about the estimate of effect).^[20]^

### 2.6 Ethical review and informed consent of patients

Ethics and dissemination: The content of this article does not involve moral approval or ethical review and will be presented in print or at relevant conferences.

## 3 Discussion

The outbreak of COVID-19 is a disaster for humanity without a doubt. Although Severe Acute Respiratory Syndrome (SARS) and Middle East Respiratory Syndrome (MERS) have caused local epidemics before, the impact of COVID-19 is clearly greater this time. It has been more than 8 months since the began of COVID-19, during which the number of daily infections and deaths people has been on the rise. In order to reduce the spread between people, many countries and regions have adopted quarantine policies, which has sharply reduced the space of former participants of exercise. Existing evidence has shown that regular exercise can significantly improve the immune capacity of the body^[21]^ and reduce the probability of infection with infectious diseases,^[22]^ which also provides a new way for the treatment of COVID-19.

This paper mainly describes how to systematically review the therapeutic effect of exercise on COVID-19, which mainly includes 4 aspects: literature collection, literature screening, data extraction and data analysis. This study can provide some methods guidance for other scholars to study the prevention and treatment effect of exercise on COVID-19 in the future. However, due to the limitation of language, we only searched Chinese and English literature, which is also the deficiency of this study.

## Author contributions

**Conceptualization:** Zhangmeng Xu.

**Data curation:** Duoduo Yu, Dongdong Mao, Donghong Feng, Tao Li.

**Formal analysis:** Zhangmeng Xu, Yong Chen.

**Funding resources:** Yaming Yu.

**Investigation:** Shengsong Yan, Ting Wang, Duoduo Yu, Donghong Feng.

**Methodology:** Zhangmeng Xu, Yong Chen, Duoduo Yu, Dongdong Mao.

**Software:** Zhangmeng Xu, Yong Chen, Yaming Yu.

**Writing – original draft:** Zhangmeng Xu.

**Writing – review & editing:** Yaming Yu, Yong Chen.

## Abbreviations

Abbreviations: CIs = confidence interval, COVID-19 = Corona Virus Disease 2019, PRISMA-P = Preferred Reporting Items for Systematic Review and Meta-Analysis Protocols, RCT = randomized controlled trial, RR = risk ratio, SARS-CoV-2 = severe acute respiratory syndrome coronavirus 2, WHO = World Health Organization.
